# Concentration- and Sequence-Dependent MRI Signal Intensity Behavior of *Ilex paraguariensis* Aqueous Extract in MRCP-like Sequences: A Preclinical Phantom Study

**DOI:** 10.3390/jimaging12070313

**Published:** 2026-07-10

**Authors:** Mario J. Noh-Burgos, Juan B. Chalé-Dzul, Leticia Olivera-Castillo, César Puerto-Castillo, Nina Méndez-Domínguez, Rosa E. Moo-Puc

**Affiliations:** 1 Hospital Regional de Alta Especialidad de la Península de Yucatan, Servicios de Salud del Instituto Mexicano del Seguro Social para el Bienestar (IMSS-BIENESTAR), Merida 97130, Yucatan, Mexico; marionohburgos17@gmail.com (M.J.N.-B.); ninamendezdominguez@gmail.com (N.M.-D.); 2 Secretaría de Ciencia, Humanidades, Tecnología e Innovación (SECIHTI), Mexico City 03940, Mexico; 3 Hospital de Especialidades, Centro Medico Nacional Ignacio García Téllez, Unidad de Investigación Medica Yucatan (UIMY), Merida 97150, Yucatan, Mexico; 4 Centro de Investigación y de Estudios Avanzados del IPN, Unidad Merida, Merida 97310, Yucatan, Mexico; leticia.olivera@cinvestav.mx (L.O.-C.); cesarp@cinvestav.mx (C.P.-C.); 5 Applied Science Research Foundation, A.C., Merida 97138, Yucatan, Mexico; 6 Ciencia y Herbolaria, Conkal 97345, Yucatan, Mexico

**Keywords:** magnetic resonance cholangiopancreatography, negative oral contrast agent, *Ilex paraguariensis* A. St.-Hil.

## Abstract

Magnetic resonance cholangiopancreatography (MRCP) is widely used for biliopancreatic imaging; however, hyperintense gastrointestinal fluids in heavily T2-weighted sequences may interfere with visualization of the biliary and pancreatic ducts. Natural manganese-containing beverages have been investigated in MRCP-related imaging contexts, and yerba mate (*Ilex paraguariensis* A. St.-Hil.) has been studied to this end. However, its concentration- and sequence-dependent signal behavior under MRCP-like phantom conditions remains insufficiently characterized. This preclinical phantom study evaluated the concentration- and sequence-dependent MRI signal intensity behavior of an aqueous extract of *Ilex paraguariensis*. The extract was characterized by means of elemental analysis, total manganese and iron quantification, total phenolic content, antioxidant capacity, and LC-ESI-MS analysis. MRI phantom experiments were run at different extract concentrations using T1-weighted, T2-weighted, and single-shot turbo spin echo (SSHTSE) sequences. The dried extract contained 1.22 ± 0.04 mg/g total manganese and 0.40 ± 0.01 mg/g total iron. Calculated total Mn concentrations in phantom dilutions ranged from 0.06 to 0.97 mg/dL. The extract showed concentration- and sequence-dependent signal behavior, with T1-weighted signal enhancement and progressive signal suppression in T2-weighted and SSHTSE sequences. No T1/T2 mapping or r1/r2 relaxivity measurements were performed. LC-ESI-MS identified MS1-based putatively assigned phenolic features without MS/MS confirmation of extract peaks. *Ilex paraguariensis* aqueous extract showed preliminary concentration- and sequence-dependent MRI signal intensity changes under phantom conditions, including signal suppression in MRCP-like heavily T2-weighted sequences. These findings do not establish clinical applicability, safety, tolerability, comparative efficacy, or improved duct visualization. Further studies are needed, incorporating relaxometric measurements, comparator agents, formulation assessment, in vivo evaluation, and clinical validation.

## 1. Introduction

Magnetic resonance cholangiopancreatography (MRCP) is a noninvasive imaging technique widely used to evaluate the biliary and pancreatic ductal systems [1,2,3,4]. It relies heavily on T2-weighted sequences in which static or slowly moving fluids appear hyperintense, allowing detailed visualization of the biliary tree and pancreatic ducts [5,6,7]. Hyperintense signals caused by gastrointestinal fluids may overlap with the pancreatobiliary anatomy and reduce diagnostic conspicuity, particularly in the duodenum and stomach [8]. To minimize these artifacts, several oral preparations have been investigated in MRCP-related studies for their ability to reduce gastrointestinal signal intensity under specific imaging conditions [3,5,6,7].

Several synthetic and natural oral contrast preparations have been investigated for MRCP applications. Among these, manganese-containing beverages have attracted attention because manganese-containing solutions may influence MRI signal intensity in a sequence-dependent manner [9,10]. Commercially available products such as blueberry juice, pineapple juice, and other manganese preparations have demonstrated varying degrees of signal suppression in heavily T2-weighted sequences [1,3,11]. Considerable heterogeneity exists regarding manganese concentration, physicochemical composition, imaging protocols, and reproducibility among studies, which limits direct comparisons and broader translational application [4,8].

*Ilex paraguariensis* A. St.-Hil., commonly known as yerba mate, is a broadleaf, evergreen shrub native to South America. In several countries, particularly in South America, it is used to prepare aqueous beverages [12,13]. Legally consumed as a natural beverage, it is commercially available as processed dried leaf products intended for preparing infusions. In addition to its dietary and cultural relevance, *I. paraguariensis* has been studied for its phytochemical composition, including phenolic compounds, flavonoids, methylxanthines, and trace metals such as manganese [14,15,16]. Previous studies have reported antioxidant, anti-inflammatory, hypolipidemic, and neurostimulant properties associated with yerba mate preparations [13,17]; these biological or nutritional properties in no way indicate its suitability for MRI contrast use.

The appeal of *I. paraguariensis* for use in the present study was its ready commercial availability, aqueous extractability, previously reported manganese-containing composition, and complex phytochemical matrix. These support its preliminary evaluation under controlled MRI phantom conditions. Any possible contribution of phenolic constituents, methylxanthines, or metal phytochemical interactions to MRI signal behavior remains hypothetical unless directly evaluated through dedicated mechanistic studies, including metal speciation, binding assays, and relaxometric measurements.

Natural manganese-containing beverages have been evaluated previously as oral negative contrast preparations, but many studies have focused primarily on qualitative or visual imaging outcomes [1,3,11]. Quantitative investigations integrating MRI signal intensity behavior with elemental and phytochemical characterization remain limited. Furthermore, concentration- and sequence-dependent MRI signal intensity behavior of aqueous *Ilex paraguariensis* extract under controlled MRCP-like phantom conditions remains insufficiently described [11]. In particular, the relationship between calculated total manganese concentration, fixed sequence MRI signal intensity measurements, and basic physicochemical features of the extract requires further preclinical characterization [18,19].

The feasibility of using an *I. paraguariensis*-based herbal extract as an oral MRI contrast preparation has been addressed [11]. The present study was designed to provide a controlled concentration- and sequence-dependent evaluation of aqueous *I. paraguariensis* extract under MRCP-like phantom conditions and to integrate this imaging behavior with elemental and basic phytochemical characterization. Specifically, serial extract concentrations, calculated total Mn concentrations, normalized signal intensity, signal enhancement or suppression percentages, and signal-to-noise ratio (SNR) were evaluated across T1-weighted, T2-weighted, and SSHTSE sequences. In parallel, total manganese and iron contents, total phenolic content, antioxidant capacity, and LC-ESI-MS MS1-based putative phenolic features were assessed to provide a basic physicochemical profile of the extract. This approach was intended to refine the preclinical characterization of *I. paraguariensis* extract under standardized phantom conditions, not to establish clinical efficacy, safety, tolerability, in vivo performance, improved duct visualization, or direct clinical applicability as a candidate preparation for future preclinical evaluation. This study was not designed to establish comparative efficacy against previously reported natural oral contrast preparations, manganese salt solutions, ferumoxsil-like preparations, or commercially available oral contrast agents. Direct comparator studies under the same MRI protocol, together with T1/T2 mapping, r1/r2 relaxivity measurements, gastric dilution models, formulation studies, safety assessment, and clinical validation, would be required before any comparison with previously evaluated natural or synthetic preparations used in MCRP-related research can be made.

## 2. Materials and Methods

### 2.1. Plant Material

Dried processed yerba mate (*Ilex paraguariensis*) was purchased in 2024 from a local commercial supplier in Merida, Yucatan, Mexico. The commercial product was a 500 g package of Rosamonte Yerba Mate (*Ilex paraguariensis*), processed containing leaves and stems (In Spanish: *Elaborada con palo*). The package label indicated to be produced and packaged by HREÑUK S.A. (Establecimiento “10 Hermanos”, Apóstoles, Argentina), and imported and distributed in Mexico by TERANA S.A. (Ciudad de México, México). It also indicated the material’s geographical origin to be Argentina (batch number L150924, expiration date September 2026). The manufacturer and distributor websites are https://rosamonte.com.ar (accessed on 26 June 2026) and https://www.terana.com (accessed on 26 June 2026), respectively. The package is described as dried and processed yerba mate leaves and stems intended for beverage preparation.

The plant material was visually inspected before extraction to confirm the absence of visible contaminants, deterioration, or foreign matter. Botanical identity was based on the package label, macroscopic characteristics of the dried material, and comparison with taxonomic descriptions. No formal botanical authentication by a taxonomist was performed. The plant material was stored in airtight containers protected from light and humidity at room temperature until extraction (approximately 2 months). Extraction was performed in 2025, and moisture content was not measured.

### 2.2. Extraction Procedure

An aqueous extract of *I. paraguariensis* was prepared by decoction using a hot aqueous extraction approach adapted from a previously reported method for *I. paraguariensis* aqueous preparations [15]. Briefly, 500 g dried processed *I. paraguariensis* from the same commercial plant material batch was subjected to three sequential decoction cycles. In each cycle, the plant material was extracted with 3 L distilled water under continuous heating at approximately 100 °C for 2 h. A total extraction volume of 9 L distilled water was used during the three extraction cycles. After each decoction cycle, the extract was cooled to room temperature and filtered through filter paper to remove insoluble plant residues. The filtrates obtained from the three extraction cycles were combined, frozen, and lyophilized to produce a dried aqueous extract. The dried extract was weighed, placed in airtight containers protected from light, and stored at 4 °C until use. Extraction yield was 17.2% (*w*/*w*), calculated from 500 g dried plant material, which yielded 86 g dried extract. Extraction reproducibility across independent plant batches was not evaluated because the material was from the same batch.

### 2.3. Elemental Analysis

Elemental analysis of the unprocessed dried *I. paraguariensis* and the corresponding aqueous extract was performed to quantify nitrogen (N) and carbon (C) contents. Samples were weighed into tin capsules and analyzed using a Flash EA 1112 elemental analyzer (Thermo Scientific/ThermoQuest, Rodano, Italy). This device operates using the dynamic flash combustion method based on the modified Dumas principle [20,21]. Carbon and nitrogen contents were quantified under standard operating conditions and expressed as percentage composition (%).

### 2.4. Total Manganese and Iron Contents

Total manganese and iron contents were measured by flame atomic absorption spectrophotometry (FAAS). Briefly, 0.5 g dried *I. paraguariensis* extract was subjected to microwave-assisted acid digestion following Environmental Protection Agency protocol EPA 3052 (MF 100-T16) [22,23]. Metal quantification was performed using an AAnalyst 800 Atomic Absorption Spectrophotometer (PerkinElmer, Inc., Shelton, CT, USA) equipped with an air–acetylene flame system. Analytical quality control was supported by the MAT REF 2676 analysis of reference material. Metal concentrations were expressed as mg of metal per g of dried extract (mg/g) and values presented as the mean of three replicates ± standard deviation (SD).

FAAS was used to determine total metal content but does not determine the free Mn^2+^ fraction, manganese oxidation state, or complex manganese species. Therefore, manganese values were reported and interpreted as total manganese content in the dried extract, whereas concentrations in phantom dilutions were reported as calculated total Mn concentrations.

### 2.5. Determination of Total Phenolic Content

Total phenolic content (TPC) was measured using Folin–Ciocalteu reagent with some modifications [24,25]. Briefly, 20 µL extract solution (5 mg/mL) was mixed with 100 µL Folin–Ciocalteu reagent (1:3, *v*/*v*), 40 µL 1 M sodium carbonate (Na_2_CO_3_) solution, and 40 µL ultrapure water in a 96-well microplate. The mixture was incubated for 80 min at room temperature in the dark and absorbance measured at 756 nm using a Varioskan LUX plate reader (Thermo Fisher Scientific, Waltham, MA, USA). A calibration curve was constructed using gallic acid (Sigma-Aldrich^®^, St. Louis, MO, USA) at concentrations ranging from 10 to 100 µg/mL. All measurements were performed in triplicate and TPC expressed as mg of gallic acid equivalents per gram of dried extract (mg GAE/g). Values were presented as the mean of three replicates ± standard deviation (SD).

### 2.6. Antioxidant Capacity

Total antioxidant capacity was quantified using the oxygen radical absorbance capacity (ORAC) assay as described by Garrett et al., with minor modifications [26,27]. Samples and Trolox standards were prepared in phosphate buffered saline (PBS, 1 mg/mL), serially diluted twofold, and 20 µL of each dilution was transferred into black 96-well microplates. Subsequently, 200 µL fluorescein stock solution (30 nmol/L) and 75 µL 2,2′-azobis(2-amidinopropane) dihydrochloride (AAPH) stock solution (12 mmol/L) were added to each well. A standard curve was produced using Trolox as the antioxidant calibrator (0–4 nmol/L). The reaction was carried out at 37 °C, and fluorescence recorded every 3.5 min for 31 cycles using an Appliskan microplate reader (Thermo Scientific, Waltham, MA, USA), with excitation and emission wavelengths set to 485 and 535 nm, respectively. Measurements were taken in triplicate for the sample and standard dilutions. ORAC was calculated from the net area under the fluorescence decay curve using the Trolox calibration curve and expressed as µmol Trolox equivalents per gram of dried extract (µmol TE/g).

### 2.7. Chemical Profiling

#### 2.7.1. High-Performance Liquid Chromatography

High-performance liquid chromatography (HPLC) analysis was run using an Agilent 1260 liquid chromatography system equipped with a quaternary pump, autosampler, and diode array detector set at 245 nm. A Hypersil GOLD C18 column (Thermo Scientific; 150 × 4.6 mm, 5 µm) was used, coupled to a Zorbax Extend C18 analytical guard column (Agilent, Santa Clara, CA, USA; 4.6 × 12.6 mm, 5 µm), and operated at a column temperature of 42 °C. The mobile phase consisted of (A) 1% (*v*/*v*) acetic acid and (B) methanol, at a flow rate of 1.2 mL/min. The following linear gradient was used: initial, 99% A for 2 min; decrease to 25% A over 18 min and hold for 5 min; increase to 99% B over 4 min and hold for 3 min; return to 99% A for 5 min; and final re-equilibration at 99% A for 10 min before the next injection. The sample injection volume was 20 µL (5 mg/mL) [28].

#### 2.7.2. Mass Spectrometry

LC-ESI-MS analysis was performed using an Agilent 1290 HPLC system equipped with a binary pump, autosampler, electrospray ionization (ESI) interface, and a low-resolution triple quadrupole (QQQ) mass analyzer. Chromatographic conditions were the same as those described in the previous section. The ESI source parameters were as follows: gas temperature, 300 °C; gas flow, 5 L/min; nebulizer pressure, 45 psi; sheath gas temperature, 250 °C; sheath gas flow, 11 L/min; capillary voltage, 3500 V; and nozzle voltage, 500 V. LC-ESI-MS data were recorded in negative ionization mode, and MS1 spectra recorded over the *m/z* range of 0–1000 [28].

Because the analysis was performed with a low-resolution QQQ mass analyzer, ion signals were evaluated using nominal *m/z* values. High-resolution accurate mass measurements were not obtained, and mass errors in ppm were not calculated. The mass tolerance parameter in the processing software was set to 0; therefore, no additional mass tolerance window was applied during data extraction. For MS1-based feature annotation in negative ionization mode, deprotonated ions ([M–H]^–^) were considered when applicable; however, adduct assignments were treated as tentative and were not used as confirmatory evidence.

MS2 acquisition was available only for selected analytical standards and/or monitored features; however, the collision energy was set to 0 eV. Therefore, no collision-induced fragmentation spectra suitable for structural confirmation were generated for the corresponding extract peaks. Feature assignment in the extract was based only on retention-time correspondence with analytical standards and nominal MS1 *m/z* signals acquired in negative ionization mode. Consequently, definitive compound identification was not attempted. The detected extract signals were classified as MS1-based, putatively assigned phenolic features rather than confirmed compounds.

### 2.8. Phantom Preparation

Phantom samples were prepared from the *I. paraguariensis* aqueous extract using a 96-well plate as an exploratory multiwell phantom platform. A stock solution of 20 mg/mL was prepared in distilled water. Appropriate aliquots of this stock solution were added directly to wells of a 96-well plate and diluted with distilled water to obtain final extract concentrations of 8, 6, 4, 2, 1, and 0.5 mg/mL in a final volume of 300 µL per well [29]. The water control consisted of 300 µL of distilled water per well and was used as the control and normalization reference.

Distilled water was selected as the control because it provides a reproducible fluid reference with high signal intensity in T2-weighted and SSHTSE sequences. The water control was used as an internal reference for signal normalization rather than as a clinical comparator. No positive comparator, such as pineapple juice, blueberry juice, manganese salt solution, ferumoxsil-like preparation, or other previously evaluated preparation, was included because this study was designed as an exploratory concentration- and sequence-dependent phantom evaluation rather than as a direct comparative efficacy study.

The 96-well plate was used as a multiwell phantom platform to allow simultaneous imaging of the extract dilutions and water controls under identical acquisition conditions. Each concentration was evaluated in two independent phantom measurements (*n* = 2). These phantom experiments were designed to evaluate concentration- and sequence-dependent signal intensity behavior under controlled conditions, not to simulate in vivo gastrointestinal dilution or clinical oral administration.

For MRI acquisition, the wells containing the extract dilutions and water controls were arranged in a fixed linear sequence and maintained in the same position across T1-weighted, T2-weighted, and SSHTSE acquisitions. The concentration sequence was kept constant for all sequences and independent measurements. The displayed order in the representative images was background noise ROI, water control, 8, 6, 4, 2, 1, and 0.5 mg/mL. The same phantom arrangement was used for quantitative ROI analysis.

### 2.9. MRI Phantom Evaluation

MRI phantom experiments were run to evaluate the concentration- and sequence-dependent signal intensity behavior of the *I. paraguariensis* aqueous extract. Signal intensities were assessed using T1-weighted, T2-weighted, and SSHTSE sequences. All MRI acquisitions were performed using a 1.5-T MRI system (MAGNETOM Amira A BioMatrix System, Siemens Healthineers, Erlangen, Germany) equipped with a multichannel phased-array receiver coil configuration consisting of a BioMatrix Body 13 with Respiratory Sensor combined with a Spine 18 coil, according to the standard institutional magnetic resonance cholangiopancreatography (MRCP) protocol. The same acquisition parameters were applied to all extract concentrations and to the water control within each sequence.

The T1-weighted sequence was acquired with repetition time (TR) = 443 ms, echo time (TE) = 8.7 ms, matrix size = 256 × 256, field of view (FOV) = 230 × 230 mm, slice thickness = 6 mm, and three acquisitions.

The T2-weighted sequence was obtained with TR = 4000 ms, TE = 102 ms, matrix size = 256 × 256, FOV = 230 × 230 mm, slice thickness = 6 mm, four acquisitions, and a TSE factor of 15.

The SSHTSE sequence was acquired with TR = 8000 ms, TE = 449 ms, matrix size = 256 × 256, FOV = 230 × 230 mm, slice thickness = 6 mm, two acquisitions, and a TSE factor of 200.

No T1 or T2 mapping, multi-TE or multi-TI relaxometric acquisition, or r1/r2 relaxivity calculations were performed. Therefore, the MRI evaluation was limited to sequence-dependent signal intensity measurements under fixed acquisition parameters.

Representative images were exported using the same display window and level settings within each MRI sequence to allow visual comparison across concentrations. No nonlinear post-processing, filtering, denoising, or contrast enhancement was applied. Image processing for figure preparation was limited to cropping, labeling, and panel arrangement. Quantitative measurements were performed on the original calibrated DICOM images in ImageJ, not on the exported figure panels.

### 2.10. ROI-Based Image Analysis

MRI signal intensity was measured using the ImageJ software (version 1.54, National Institutes of Health, Bethesda, MD, USA). Circular regions of interest (ROIs) of identical size were manually placed within the central portion of each phantom well [4]. The same ROI size was used across all phantom samples within each independent measurement and sequence; only the ROI position was adjusted to center the ROI within each phantom sample. ROIs were placed to avoid container borders, air-liquid interfaces, visible artifacts, and partial volume effects. Representative ROI placement, including phantom ROIs and a background noise ROI, was used for quantitative image analysis.

The spatial calibration available from the DICOM image properties in ImageJ was used for ROI area reporting. ROI areas were measured from the calibrated image properties and converted from µm^2^ to mm^2^ when applicable. These areas were kept comparable between the two independent measurements, with mean ROI areas of 26.87 ± 0.83 mm^2^ for T1-weighted images, 22.34 ± 0.28 mm^2^ for T2-weighted images, and 22.60 ± 0.00 mm^2^ for SSHTSE images.

Two independent phantom measurements were analyzed for each extract concentration and the water control (*n* = 2). Mean signal intensity was obtained from the “Mean” value reported by ImageJ for each phantom ROI. Multiple water control ROIs were measured at position-matched locations and used as internal normalization references for the corresponding extract concentrations.

Background noise was estimated using a separate ROI placed in an artifact-free background region outside the phantom well, avoiding ghosting artifacts, wrap-around artifacts, container borders, and regions affected by visible background inhomogeneity. The standard deviation of the background ROI signal was used as the noise estimate. A single background noise SD value was used for all phantom wells within the same sequence and independent measurement.

The signal-to-noise ratio (SNR) was calculated using the following formula:

SNR = mean signal intensity of phantom ROI/standard deviation of background noise ROI.

The SNR of the water control was calculated using the same formula. In the revised analysis, position-matched water SNR values were also reported to avoid assigning “NA” to the water control.

Normalized signal intensity was calculated by dividing the mean signal intensity of each extract concentration by the mean signal intensity of the position-matched water control acquired with the same sequence and independent measurement:

Normalized signal intensity = mean SI of extract concentration/mean SI of position-matched water control.

For T1-weighted images, signal enhancement (%) was calculated as:

Signal enhancement (%) = [(normalized signal intensity − 1) × 100].

For T2-weighted and SSHTSE images, signal suppression (%) was calculated as:

Signal suppression (%) = [(1 − normalized signal intensity) × 100].

Raw ROI signal intensity, position-matched water control values, background noise SD, normalized signal intensity, signal enhancement or suppression percentage, SNR, and water SNR values are provided separately in Supplementary Tables S1–S3.

Because signal intensity in phantom images may be affected by spatial variations related to coil sensitivity, field inhomogeneity, and container position within the imaging field, quantitative measurements were taken using standardized ROIs placed within the central portion of each phantom well, avoiding container borders and visible artifacts. Position-matched water controls were used to reduce the influence of spatial signal variation on normalized signal intensity calculations. Nevertheless, residual spatial signal variation across the image field remains a limitation of signal intensity-based phantom analysis.

### 2.11. Statistical Analysis

Quantitative MRI data were analyzed descriptively using GraphPad Prism (version 9.0, GraphPad Software, San Diego, CA, USA). Signal intensity values were normalized relative to the water control, which served as a reference for signal normalization rather than as a clinical comparator. Signal enhancement, signal suppression percentages, signal-to-noise ratio (SNR), and coefficient of variation (CV%) were calculated for each concentration when applicable.

Coefficient of variation (CV%) was calculated as SD/mean × 100 using normalized signal intensity values for each concentration. CV values were interpreted descriptively because the phantom experiments involved only two independent measurements (*n* = 2).

Results were presented as mean ± standard deviation (SD). Because of the limited number of independent measurements, no inferential statistical comparisons, ANOVA, regression modeling, confidence interval estimation, or formal modeling of concentration-associated patterns were performed. Therefore, the observed trends were interpreted descriptively and should be considered exploratory.

## 3. Results

### 3.1. Elemental Composition of Ilex paraguariensis Leaves and Extract

Elemental analysis identified compositional differences between the raw dried *I. paraguariensis* leaves and the corresponding extract, including changes in nitrogen and carbon content after aqueous extraction and lyophilization (Table 1). Variations in the C:N ratio were also observed between the raw plant material and the extract.

### 3.2. Quantification of Total Manganese and Iron in Ilex paraguariensis Extract

Total manganese and iron contents in the *I. paraguariensis* aqueous extract were measured by flame atomic absorption spectrophotometry (FAAS). Manganese was detected at a higher concentration than iron in the dried aqueous extract (Table 1). Analytical quality control was supported by the MAT REF 2676 analysis of reference material. The measured total manganese content in the dried extract was subsequently used to calculate the total Mn concentrations corresponding to the different extract dilutions evaluated in the MRI phantom experiments.

### 3.3. Total Phenolic Content

The total phenolic content (TPC) of the *I. paraguariensis* aqueous extract was measured using the Folin–Ciocalteu colorimetric method. The extract had a high total phenolic content (mg GAE/g, Table 1). These results are consistent with the presence of abundant phenolic constituents in the aqueous extract.

### 3.4. Antioxidant Capacity (ORAC Assay)

The antioxidant capacity of the *I. paraguariensis* aqueous extract, quantified by the ORAC assay (µmol TE/g), was high (Table 1). These results provide physicochemical information about the extract but should not be interpreted as direct evidence of MRI contrast performance.

### 3.5. Chemical Profiling via HPLC-ESI-MS

**Figure 1:**
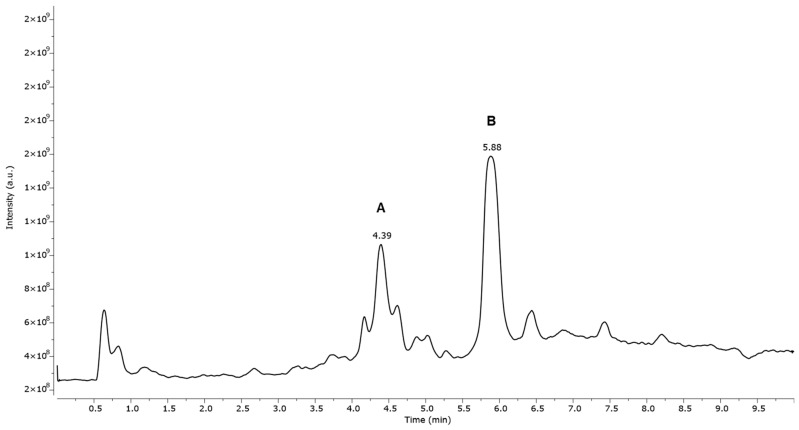
Total ion chromatogram (TIC) of the *Ilex paraguariensis* aqueous extract, produced by LC-ESI-MS. Two major chromatographic regions, A and B, were detected at retention times of approximately 4.39 min and 5.88 min, respectively, corresponding to predominant polar features detected under the chromatographic conditions used.

Liquid chromatography-electrospray ionization–mass spectrometry (LC-ESI-MS) analysis of the *I. paraguariensis* aqueous extract identified two main chromatographic regions: Region A (Rt ≈ 4.01–4.70 min) and Region B (Rt ≈ 5.60–6.10 min) (Figure 1).

Retention-time comparison with analytical standards showed that rutin eluted at Rt ≈ 4.02 min, whereas quercetin eluted at Rt ≈ 6.07 min under the chromatographic conditions used. Based primarily on this retention-time correspondence, Regions A and B were considered for putative phenolic feature annotation.

In Region A, the extract MS1 spectrum acquired at Rt 4.20 min exhibited nominal signals at *m*/*z* ≈ 300.97, 609.91, and 621.30. The signal at *m*/*z* ≈ 609.91 is compatible with a rutin-related ion, possibly corresponding to the expected nominal deprotonated molecule [M−H]^−^, whereas the signal at *m*/*z* ≈ 300.97 may correspond to a quercetin aglycone-related ion potentially generated by in-source fragmentation of a rutin-like glycosylated flavonoid. In addition, the nominal signal at *m*/*z* ≈ 621.30 was observed in the extract and in the rutin analytical standard. In the latter, the signal at *m*/*z* 621.30 was selected as the precursor ion for reference MS/MS acquisition and yielded a reference ion at *m*/*z* 609.27 (Figure S1). However, because the adduct identity of the *m*/*z* 621.30 signal was not established and MS/MS fragmentation was not acquired for the corresponding extract peak, these signals should be interpreted as descriptive MS1 evidence supporting a rutin-like putative phenolic feature rather than definitive identification of rutin or its fragments in the extract.

In Region B, the extract MS1 spectrum acquired at Rt 5.90 min showed nominal signals in the *m*/*z* 300–310 range, including one around *m*/*z* ≈ 307.30. This chromatographic region exhibited retention-time correspondence with the quercetin analytical standard. For the quercetin standard, the nominal precursor ion at *m*/*z* 307.30 was selected and yielded a reference ion at *m*/*z* 301.51 (Figure S2). However, this MS/MS information was obtained only from the analytical standard and not from the corresponding extract peak. Therefore, the Region B feature is reported as a quercetin-like putative phenolic feature based primarily on retention-time correspondence and descriptive MS1 information, rather than as definitive identification of quercetin in the extract.

Because the LC-ESI-MS analysis was run using a low-resolution triple quadrupole mass analyzer, the detected signals were interpreted using nominal *m*/*z* values. High-resolution accurate mass measurements, isotope-pattern confirmation, and mass-error calculations in ppm were not available. Adduct assignments were therefore not established for the extract signals. In addition, because MS/MS fragmentation was not acquired for the corresponding extract peaks, compound-level identification could not be established.

A summary of the detected LC-ESI-MS features and their putative annotations is provided in Table 2. Detailed LC-ESI-MS chromatographic profiles, representative MS1 spectra of the extract at selected retention times, and reference MS/MS spectra of analytical standards are provided in the Supplementary Material (Figures S1–S4).

### 3.6. Sequence-Dependent MRI Signal Intensity Evaluation in Phantom Condition

**Figure 2:**
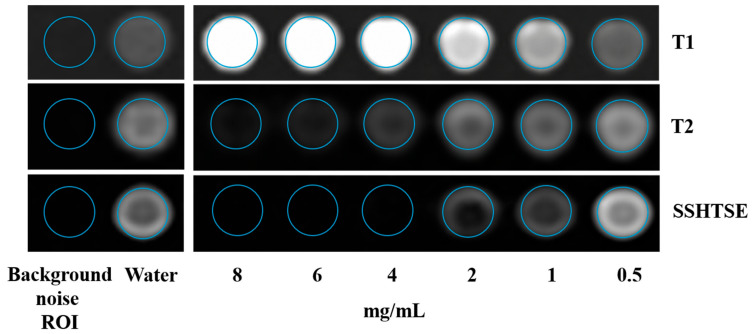
Representative MRI phantom images acquired using a 96-well plate arrangement. Rows correspond to T1-weighted, T2-weighted, and SSHTSE images. The wells were arranged in a fixed linear sequence corresponding to the background noise ROI, water control, and *Ilex paraguariensis* aqueous extract concentrations of 8, 6, 4, 2, 1, and 0.5 mg/mL. Circular ROIs were placed within the central portion of each well, avoiding well borders, air-liquid interfaces, visible artifacts, and partial volume effects. A separate background ROI was placed outside the phantom wells for noise estimation. Representative images were exported using the same window and level settings within each sequence. Image processing for figure preparation was limited to cropping, labeling, and panel arrangement.

The *I. paraguariensis* aqueous extract exhibited concentration- and sequence-dependent changes in MRI signal intensity across the evaluated acquisition protocols. As shown in Figure 2, signal enhancement was observed in T1-weighted images, whereas a progressive decrease in signal intensity was observed in T2-weighted and SSHTSE sequences as extract concentration increased. These results reflect signal intensity behavior under fixed sequence parameters and should not be interpreted as direct relaxometric measurements.

The corresponding calculated total Mn concentrations for each extract dilution were estimated from the total manganese content measured in the dried extract (Table 3). Calculated total Mn concentrations ranged from 0.06 to 0.98 mg/dL across the evaluated extract concentrations. Higher extract concentrations corresponded to higher calculated total Mn concentrations and greater signal suppression in T2-weighted and SSHTSE sequences.

Based on the signal behavior observed in the phantom experiments, quantitative signal intensity analysis was performed using regions of interest (ROIs) defined in the ImageJ software. ROI-based measurements were used to describe apparent concentration-related and sequence-dependent signal intensity changes across the different MRI acquisition protocols.

**Figure 3:**
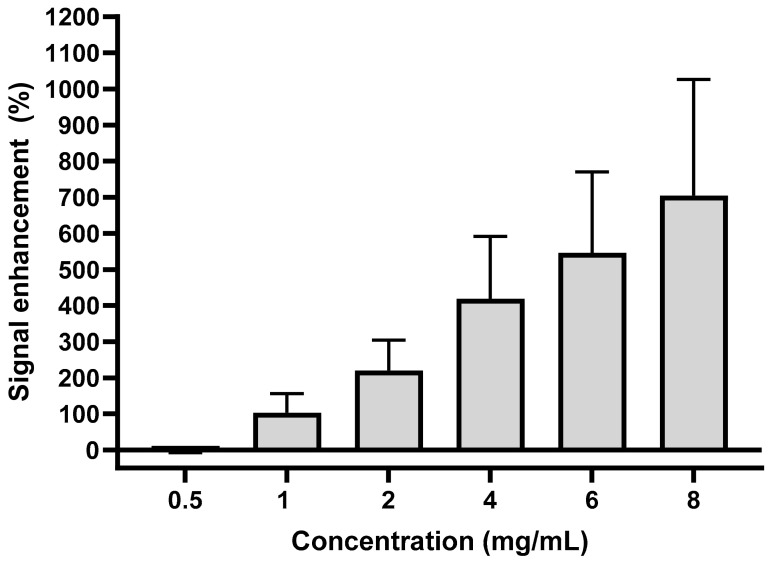
Signal enhancement (%) as a function of extract concentration in T1-weighted MRI images. Values were normalized relative to the water control, which served as the 0% baseline. Data are presented as mean ± standard deviation (SD) from two independent measurements (*n* = 2).

In T1-weighted images, an apparent concentration-related increase in signal intensity was observed as extract concentration increased (Figure 3). This descriptive trend is summarized in Table 4, where normalized signal intensity increased from 0.98 ± 0.06 at 0.5 mg/mL to 8.04 ± 3.22 at 8 mg/mL. The highest extract concentration evaluated, 8 mg/mL, showed the greatest signal enhancement. These findings should be interpreted descriptively because only two independent phantom measurements were performed.

**Figure 4:**
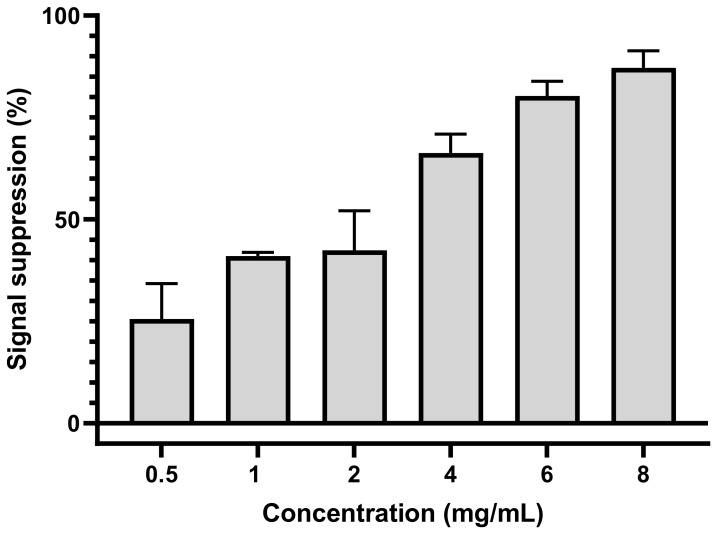
Signal suppression (%) as a function of extract concentration in T2-weighted MRI images. Values were normalized relative to the water control, which served as the 0% baseline. Data are presented as mean ± standard deviation (SD) from two independent measurements (*n* = 2).

In contrast to the behavior observed in T1-weighted images, the T2-weighted images exhibited an apparent concentration-related decrease in signal intensity as extract concentration increased (Figure 4). Signal suppression ranged from 25.59 ± 8.67% at 0.5 mg/mL to 87.17 ± 4.18% at 8 mg/mL (Table 5). Higher extract concentrations were associated with lower signal intensity, with the lowest normalized signal intensity values observed at 6 and 8 mg/mL. These results represent sequence-dependent signal intensity changes under fixed acquisition parameters rather than direct T2 relaxivity measurements.

**Figure 5:**
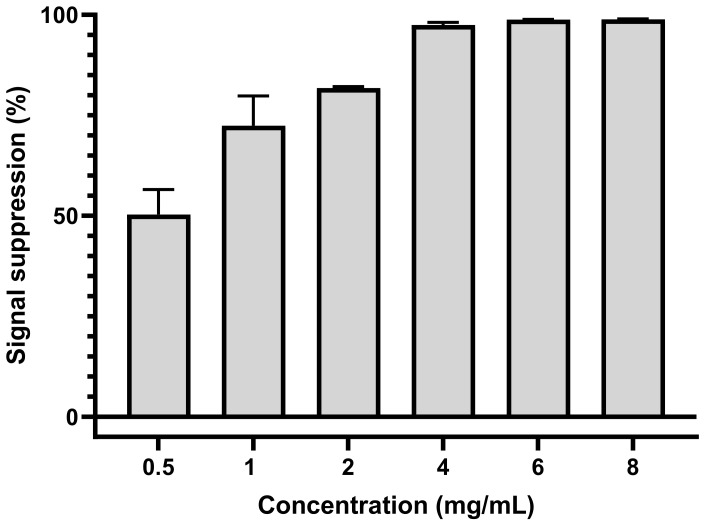
Signal suppression (%) as a function of extract concentration in heavily T2-weighted SSHTSE MRI sequences. Values were normalized relative to the water control, which served as the 0% baseline. Data are presented as mean ± standard deviation (SD) from two independent measurements (*n* = 2).

In heavily T2-weighted SSHTSE sequences, an apparent concentration-related decrease in signal intensity was observed as extract concentration increased (Figure 5). Suppression levels exceeding 97% were observed at concentrations ≥ 4 mg/mL (Table 6). At the highest concentrations evaluated, marked attenuation of signal intensity was observed under the fixed SSHTSE acquisition parameters used in this phantom experiment. These findings should be interpreted as descriptive signal intensity measurements and not as formal relaxometric characterization.

In addition to changes in signal intensity, SNR was evaluated descriptively to assess image quality across the different extract concentrations. SNR behavior was sequence dependent. In the T1-weighted images, SNR increased as extract concentration increased, consistent with the observed signal enhancement in this sequence. In contrast, in the T2-weighted and SSHTSE images, SNR progressively decreased at higher extract concentrations, following the attenuation of signal intensity produced by the extract. Despite this decrease, phantom structures remained visible across all evaluated concentrations.

CV% values were calculated from normalized signal intensity measurements to provide a descriptive estimate of relative variability across concentrations. The highest CV values were observed in the T1-weighted images at higher extract concentrations, consistent with the relatively large standard deviations observed in this sequence. In the T2-weighted and SSHTSE images, CV values varied across concentrations and should be interpreted cautiously, particularly at very low normalized signal intensities, where small absolute changes may produce relatively high CV values.

An apparent concentration-related trend was observed across the evaluated concentrations, with increasing extract concentrations associated with sequence-dependent MRI signal intensity changes. Given the exploratory nature of the phantom experiments and the limited number of independent measurements (*n* = 2), these findings were interpreted descriptively and should not be considered evidence of a statistically validated dose–response model.

## 4. Discussion

Aqueous extracts of *Ilex paraguariensis* exhibited concentration- and sequence-dependent MRI signal intensity changes under controlled phantom conditions. These changes were characterized by progressive signal enhancement in the T1-weighted images and marked signal suppression in the heavily T2-weighted and SSHTSE sequences. This descriptive signal behavior is consistent with previous reports describing signal attenuation by manganese-containing beverages in MRCP-related imaging contexts [7,8,9,10,11]. However, because this study was performed exclusively under phantom conditions, the findings should be interpreted as preclinical signal-intensity data and not as evidence of clinical efficacy, in vivo performance, safety, tolerability, or direct applicability to patient MRCP examinations.

The present findings should be considered in relation to the previous work by Nestle et al., who evaluated an *I. paraguariensis*/yerba mate-based herbal extract as an oral gastrointestinal MRI contrast preparation [11]. In that study, *I. paraguariensis* exhibited biphasic contrast behavior, with T1-weighted effects at short echo times and T2-weighted effects at echo times greater than approximately 40 ms. The authors proposed that the observed MRI signal behavior was related to a low-molecular-weight manganese complex based on elemental analysis, NMR relaxometry, and ESR spectroscopy; they also reported preliminary imaging results taken in staff and patient volunteers after ingestion of an instant *I. paraguariensis* preparation.

These previous findings provide an important basis for the present study. Accordingly, the current study was designed as a complementary preclinical evaluation focused specifically on the concentration- and sequence-dependent MRI signal intensity behavior of an *I. paraguariensis* aqueous extract under MRCP-like phantom conditions, rather than as a general feasibility study of *I. paraguariensis* as an oral MRI contrast preparation. In this context, the present results complement previous observations by evaluating serial extract concentrations, calculated total Mn concentrations, normalized signal intensity, signal enhancement or suppression percentages, and SNR across T1-weighted, T2-weighted, and SSHTSE sequences.

The MRI signal behavior observed here was accompanied by elemental characterization showing measurable total manganese and iron in the dried aqueous extract. Increasing extract concentration was associated with higher calculated total Mn concentrations and progressive signal changes across the evaluated sequences. However, FAAS quantified total elemental manganese and iron only and did not determine free Mn^2+^ fraction, manganese speciation, oxidation state, or the presence of metal–ligand complexes [11,30]. In addition, the present study did not include T1/T2 mapping, multi-TE or multi-TI relaxometric acquisitions, or r1/r2 relaxivity measurements [5,6]. Therefore, the data should be interpreted as descriptive signal-intensity findings under fixed acquisition parameters, rather than as formal relaxometric characterization of the extract.

The extract also had a complex phytochemical profile, as suggested by its high total phenolic content, antioxidant capacity, and LC-ESI-MS MS1-based putative phenolic features detected in chromatographic regions corresponding to the retention times of rutin and quercetin analytical standards. However, because MS/MS fragmentation was not acquired for the corresponding extract peaks, these signals should not be interpreted as definitive compound identifications [31,32,33]. In the present study, total phenolic content, antioxidant capacity, and MS1-based LC-ESI-MS features should be considered physicochemical descriptors of the extract rather than evidence of MRI contrast efficacy, clinical advantage, or a confirmed contribution to the observed signal intensity changes.

The coexistence of total manganese and phenolic constituents may be relevant for future studies evaluating metal-phytochemical interaction [30]. This interpretation is consistent with the hypothesis raised by Nestle et al., who proposed that manganese in *I. paraguariensis* may be associated with natural complexing agents [11]. Nevertheless, the present study did not directly evaluate manganese-phenolic complex formation, manganese binding, metal–ligand interactions, or phenolic-mediated changes in relaxivity. Therefore, any possible contribution of phenolic constituents to manganese availability or MRI signal behavior remains hypothetical and should be addressed in future studies using approaches such as ultrafiltration, electron paramagnetic resonance spectroscopy, metal-binding assays, speciation analysis, and relaxometric measurements.

Compared with previous studies evaluating natural oral contrast preparations for MRCP [3,4,11], the present study provides an integrated preclinical approach combining elemental analysis, basic phytochemical characterization, and quantitative MRI phantom evaluation. Earlier investigations frequently relied on qualitative image interpretation, visual grading systems, or in vivo feasibility observations, whereas the present study incorporated normalized signal intensity, enhancement or suppression percentages, SNR evaluation, and calculated total Mn concentrations under standardized phantom conditions. Nonetheless, direct comparisons among studies remain limited because of substantial heterogeneity in MRI acquisition protocols, extract preparation methods, manganese concentrations, image analysis strategies, and replicate structure [8,18,29].

A major limitation of the present study is the absence of direct comparator agents evaluated under the same MRI protocol. Although pineapple juice, blueberry juice, manganese-containing preparations, manganese salt solutions, ferumoxsil-like preparations, and other preparation have been previously evaluated in MRCP-related imaging context, none of these were included in the present phantom experiments. Therefore, the observed signal suppression cannot be interpreted as evidence of superiority, equivalence, or comparable performance relative to previously evaluated natural or synthetic preparations. Future head-to-head phantom studies using the same MRI system, acquisition parameters, concentration ranges, ROI strategy, and replicating structure will be required to determine how *I. paraguariensis* extracts compare to accepted comparator agents.

Signal suppression in the heavily T2-weighted and SSHTSE sequences increased with extract concentration, with the highest suppression values observed at the highest concentrations evaluated. However, in the SSHTSE images, signal suppression appeared to approach a plateau at concentrations ≥ 4 mg/mL, suggesting that the apparent concentration-related signal pattern may not be strictly linear across all concentration ranges. Similar nonlinear behavior has been described for previously evaluated oral contrast preparations [4,29]. Because the present study included a limited number of concentration points and only two independent measurements (*n* = 2), the observed descriptive concentration-associated pattern should be considered exploratory and should not be interpreted as a statistically validated dose–response model.

From a translational perspective, the concentration range evaluated in the present phantom study should be interpreted cautiously. Previous MRCP studies using natural manganese-containing preparations, such as pineapple juice, have shown that gastroduodenal signal suppression may depend not only on the initial preparation manganese concentration but also on dilution by gastric and duodenal fluids, timing of administration, and in vivo distribution. Therefore, the manganese content measured in the present extract may be useful only for designing future preclinical formulation and dilution studies. However, the present study did not evaluate gastric dilution, administered volume, solubility at higher concentrations, pH-dependent stability, osmolality, viscosity, palatability, safety, or in vivo MRCP performance. Consequently, no clinically applicable dose, administration volume, formulation, or clinical use recommendation can be proposed from the present phantom data.

Safety considerations are essential before any clinical translation can be proposed. Although *I. paraguariensis* is commercially available and widely consumed as a natural beverage, this does not establish its suitability as a candidate preparation for future preclinical or clinical evaluation in an MRI-related context. In addition to manganese exposure, *I. paraguariensis* contains methylxanthines such as caffeine and related compounds; caffeine, theobromine, and other xanthines were not quantified in the present extract. Therefore, the potential stimulant load, contraindications, and tolerability in sensitive populations could not be assessed. Future research should quantify methylxanthines, evaluate palatability and gastrointestinal tolerability, and assess safety in clinically relevant populations, particularly in patients with caffeine sensitivity, cardiovascular disease, pregnancy, hepatic or renal impairment, or altered manganese handling.

Several additional limitations should be acknowledged. First, the phantom setting did not reproduce key in vivo physiological conditions that may influence the observed MRI signal behavior, including the presence of bile, gastrointestinal dilution, gastric pH, gastric emptying, intestinal transit time, interactions with physiologic fluids, or actual visualization of the biliary and pancreatic ducts during clinical MRCP [8,11]. Second, the study included only two independent phantom measurements across the evaluated groups, which limits statistical power, variance estimation, confidence interval calculation, regression-based modeling of concentration-associated patterns, ANOVA-based comparisons, and robust reproducibility assessment. This limitation is particularly relevant for the T1-weighted data, where relatively high variability was observed at higher extract concentrations. Third, signal-intensity measurements may have been affected by spatial variations related to coil sensitivity, field inhomogeneity, and container position within the imaging field. Although standardized ROIs were placed within the central portion of each phantom well, residual spatial signal variation remains an inherent limitation of signal-intensity-based phantom analysis.

Plant material traceability also represents a limitation. The present study used commercially processed yerba mate material (*I. paraguariensis*) containing leaves and stems, and information regarding harvest period, post-harvest processing, pre-purchase storage history, and environmental growing conditions was limited to that available from the commercial label and supplier-provided information. No voucher specimen was deposited in a recognized herbarium because the study used a processed commercial food-grade product rather than freshly collected plant material suitable for herbarium deposition, which limits botanical traceability and reproducibility. Future studies should include botanically authenticated material, voucher specimen deposition, detailed batch documentation, moisture content determination, and extraction reproducibility across independent plant batches.

Despite its limitations, the present study contributes quantitative physicochemical and phantom MRI data supporting further preclinical investigation of *I. paraguariensis* extract as a candidate preparation for future preclinical evaluation. The integration of elemental analysis, basic phytochemical profiling, and MRI signal evaluation provides a useful framework for future studies exploring natural contrast preparations under controlled experimental conditions. However, the possible translational relevance of *I. paraguariensis* extract in MRCP remains unproven and should only be considered after relaxometric characterization, standardized extraction protocols, direct comparator studies, gastric dilution and formulation assessment, in vivo imaging studies, safety and tolerability evaluation, and prospective clinical validation in patients undergoing MRCP.

## 5. Conclusions

In this preclinical phantom study, aqueous extracts of *Ilex paraguariensis* A. St.-Hil. showed preliminary concentration and sequence-dependent MRI signal intensity changes, characterized by signal enhancement in T1-weighted sequences and signal suppression in heavily T2-weighted and SSHTSE sequences under fixed acquisition parameters. These findings were accompanied by measurable total manganese and iron contents, high total phenolic content, antioxidant capacity, and MS1-based putative phenolic features detected via LC-ESI-MS analysis.

The present results should be interpreted solely as initial phantom-based evidence and do not establish clinical applicability, in vivo performance, patient tolerability, safety, comparative efficacy, or improved visualization of the pancreatobiliary ducts during MRCP. Rather than supporting clinical use, this study provides an exploratory methodological framework integrating MRI phantom evaluation with elemental, physicochemical, and putative phytochemical characterization.

Importantly, the observed signal changes should be interpreted as sequence-dependent signal intensity changes under fixed acquisition parameters, rather than as direct evidence of T1/T2 relaxivity behavior. Further studies incorporating T1/T2 mapping, r1/r2 relaxivity measurements, standardized extraction methods, direct comparator experiments, gastric dilution models, formulation assessment, in vivo imaging, safety and tolerability evaluation, and prospective clinical validation are required to clarify the reproducibility of these findings and, only after further validation, their possible translational relevance.

## Figures and Tables

**Table 1:** Physicochemical, elemental, and antioxidant characterization of *Ilex paraguariensis* leaf material and dried aqueous extract.

Parameter			Result
Elemental composition	Leaf	Nitrogen	2.46%
		Carbon	49.80%
	Extract	Nitrogen	3.90%
		Carbon	57.13%
Metal content	Manganese		1.22 ± 0.04 mg/g
	Iron		0.40 ± 0.01 mg/g
Total phenolic content (TPC)			627.33 ± 7.51 mg GAE/g
Antioxidant capacity (ORAC)			2767.50 µmol TE/g

Values are expressed as mean ± standard deviation (SD) when replicate-derived final values were available. Nitrogen and carbon contents are reported as percentage (%) values. Manganese and iron contents are expressed as mg/g of dried extract and were measured by FAAS in triplicate (*n* = 3). Total phenolic content (TPC) is expressed as mg gallic acid equivalents per gram of dried extract (mg GAE/g) and was measured in triplicate (*n* = 3). Antioxidant capacity determined by ORAC assay was calculated from the Trolox calibration curve and expressed as µmol Trolox equivalents per gram of dried extract (µmol TE/g). Elemental composition was determined in raw leaf material and dried extract, whereas metal content, TPC, and ORAC were quantified in the dried aqueous extract.

**Table 2:** MS1-based putative phenolic features detected in the *I. paraguariensis* aqueous extract by LC-ESI-MS.

Region	Extract Rt (min)	MS1 Rt (min)	Rt-Matched Standard	Nominal *m*/*z* in Extract	Standard Reference Ion	Rt-Based Putative Annotation
A	4.01–4.70	4.20	Rutin: 4.02	300.97, 609.91, 621.30	609.27	Rutin-like phenolic feature
B	5.60–6.10	5.90	Quercetin: 6.07	307.30	301.51	Quercetin-like phenolic feature

Rt: retention time; *m*/*z*: mass-to-charge ratio; MS1: full-scan mass spectrometry. Feature annotation was based primarily on retention-time correspondence with analytical standards. Nominal MS1 signals are reported as descriptive supporting information only. Adduct assignments were not established for the extract signals. Standard reference ions were observed only in analytical standards and not in the corresponding extract peaks; therefore, these features should be interpreted as Rt-based putative annotations rather than definitive compound identifications.

**Table 3:** Calculated total Mn concentrations (mg/dL) correspond to different dilutions of the *Ilex paraguariensis* aqueous extract used in MRI phantom experiments.

Concentration mg/mL	Calculated Total Mn (mg/dL)
Water (control)	0.00
8.0	0.97
6.0	0.73
4.0	0.48
2.0	0.24
1.0	0.12
0.5	0.06

Calculated total Mn concentrations were estimated from the total manganese content measured in the dried extract. The water control contained no extract derived from manganese.

**Table 4:** Normalized signal intensity, signal enhancement (%), signal-to-noise ratio (SNR), and coefficient of variation (CV%) of normalized signal intensity in T1-weighted MRI images at different concentrations of the *Ilex paraguariensis* aqueous extract.

Concentration (mg/mL)	Normalized Signal Intensity	Signal Enhancement (%)	Signal-to-Noise Ratio (SNR)	CV (%)
Water (control)	1.00 ± 0.00	0.00 ± 0.00	28.41 ± 2.91	0.00
0.5	0.98 ± 0.06	−1.99 ± 5.55	35.05 ± 0.13	6.12
1.0	2.04 ± 0.52	103.59 ± 52.18	65.26 ± 13.14	25.49
2.0	3.20 ± 0.85	219.83 ± 84.84	90.07 ± 15.82	26.56
4.0	5.19 ± 1.72	418.92 ± 172.46	131.79 ± 27.67	33.14
6.0	6.47 ± 2.24	546.51 ± 223.96	158.45 ± 35.69	34.62
8.0	8.04 ± 3.22	704.26 ± 322.05	176.19 ± 35.93	40.05

Values are expressed as mean ± standard deviation (SD) from two independent phantom measurements (*n* = 2). CV% was calculated from normalized signal intensity values. SNR was calculated as the mean signal intensity of each phantom ROI divided by the standard deviation of a background noise ROI. The water control SNR was calculated using the same formula applied to the extract samples; position-matched water SNR values were first averaged within each independent measurement and then summarized as mean ± SD across the two measurements. Raw ROI signal intensity values, position-matched water control values, background noise SD values, normalized signal intensity values, and SNR calculations are provided in Table S1.

**Table 5:** Normalized signal intensity, signal suppression (%), signal-to-noise ratio (SNR), and coefficient of variation (CV%) of normalized signal intensity in T2-weighted MRI images at different concentrations of the *Ilex paraguariensis* aqueous extract.

Concentration (mg/mL)	Normalized Signal Intensity	Signal Suppression (%)	Signal-to-Noise Ratio (SNR)	CV (%)
Water (control)	1.00 ± 0.00	0.00 ± 0.00	297.39 ± 235.06	0.00
0.5	0.74 ± 0.09	25.59 ± 8.67	251.50 ± 159.84	12.16
1.0	0.59 ± 0.01	41.07 ± 0.87	197.84 ± 142.13	1.69
2.0	0.58 ± 0.10	42.45 ± 9.68	169.17 ± 100.08	17.24
4.0	0.34 ± 0.05	66.31 ± 4.60	94.50 ± 65.56	14.71
6.0	0.20 ± 0.04	80.30 ± 3.59	51.69 ± 35.90	20.00
8.0	0.13 ± 0.04	87.17 ± 4.18	29.12 ± 18.17	30.77

Values are expressed as mean ± standard deviation (SD) from two independent phantom measurements (*n* = 2). CV% was calculated from normalized signal intensity values. Signal suppression and SNR were calculated as described in Section 2. The water control SNR was calculated using the same approach applied to the extract samples; position-matched water SNR values were first averaged within each independent measurement and then summarized as mean ± SD across the two measurements. Raw ROI signal intensity values, position-matched water control values, background noise SD values, normalized signal intensity values, signal suppression values, and SNR calculations are provided in Table S2.

**Table 6:** Normalized signal intensity, signal suppression (%), signal-to-noise ratio (SNR), and coefficient of variation (CV%) of normalized signal intensity in heavily T2-weighted single-shot turbo spin echo (SSHTSE) images at different concentrations of the *Ilex paraguariensis* aqueous extract.

Concentration (mg/mL)	Normalized Signal Intensity	Signal Suppression (%)	Signal-to-Noise Ratio (SNR)	CV (%)
Water (control)	1.00 ± 0.00	0.00 ± 0.00	264.04 ± 99.63	0.00
0.5	0.50 ± 0.06	50.32 ± 6.26	172.23 ± 34.83	12.00
1.0	0.28 ± 0.07	72.43 ± 7.49	89.57 ± 50.29	25.00
2.0	0.18 ± 0.00	81.82 ± 0.38	50.35 ± 15.20	0.00
4.0	0.02 ± 0.01	97.51 ± 0.65	6.26 ± 3.81	50.00
6.0	0.01 ± 0.00	98.81 ± 0.10	2.49 ± 0.99	0.00
8.0	0.01 ± 0.00	98.88 ± 0.14	2.04 ± 0.90	0.00

Values are expressed as mean ± standard deviation (SD) from two independent phantom measurements (*n* = 2). CV% was calculated from normalized signal intensity values. Signal suppression and SNR were calculated as described in Section 2. The water control SNR was calculated using the same approach applied to the extract samples; position-matched water SNR values were first averaged within each independent measurement and then summarized as mean ± SD across the two measurements. Raw ROI signal intensity values, position-matched water control values, background noise SD values, normalized signal intensity values, signal suppression values, and SNR calculations are provided in Table S3.

## Data Availability

The original contributions presented in this study are included in the article/Supplementary Material. Raw MRI ROI-based data, including signal intensity values, position-matched water control values, background noise SD values, normalized signal intensity values, signal enhancement or suppression values, and SNR calculations, are provided in Supplementary Tables S1–S3. Additional data, including replicate data from physicochemical and chemical analyses, are available from the corresponding author upon reasonable request.

## Supplementary Materials

The following supporting information can be downloaded at https://www.mdpi.com/article/10.3390/jimaging12070313/s1. Table S1: Raw ROI signal intensity, position-matched water control values, background noise SD, normalized signal intensity, signal enhancement, and SNR calculation for T1-weighted images of the *Ilex paraguariensis* aqueous extract; Table S2: Raw ROI signal intensity, position-matched water control values, background noise SD, normalized signal intensity, signal suppression, and SNR calculation for T2-weighted images of the *Ilex paraguariensis* aqueous extract; Table S3: Raw ROI signal intensity, position-matched water control values, background noise SD, normalized signal intensity, signal suppression, and SNR calculation for SSHTSE images of the *Ilex paraguariensis* aqueous extract; Figure S1: LC–ESI–MS/MS analysis of rutin analytical standard; Figure S2: LC-ESI-MS/MS analysis of quercetin analytical standard; Figure S3: MS1 spectrum of the extract at Rt ≈ 4.20 min (Region A); Figure S4: MS1 spectrum of the extract at Rt ≈ 5.90 min (Region B).
