# Supplementary material for: Concentration- and Sequence-Dependent MRI Signal Intensity Behavior of Ilex paraguariensis Aqueous Extract in MRCP-like Sequences: A Preclinical Phantom Study
Source: J Imaging. 2026 Jul 10;12(7):313. doi: 10.3390/jimaging12070313 (PMC13412747; doi:10.3390/jimaging12070313)
Supplement: Supplementary file 1 [file jimaging-12-00313-s001.zip › jimaging-4360458-supplementary.pdf]

Supplementary Material

Mario J. Noh-Burgos [1,2], Juan B. Chalé-Dzul [3], Leticia Olivera-Castillo [4,5], César A. Puerto-Castillo [4], Nina Méndez-Domínguez [1], Rosa E. Moo-Puc [1,2,6]

Table S1. Raw ROI signal intensity, position-matched water control values, background noise SD, normalized signal intensity, signal enhancement and SNR calculation for T1-weighted images of the *Ilex paraguariensis* aqueous extract.

Conc. (mg/mL)	Raw mean SI M1	Raw mean SI M2	Water mean SI M1	Water mean SI M2	Background noise SD M1	Background noise SD M2	Normalized SI M1	Normalized SI M2	Signal enhancement M1 (%)	Signal enhancement M2 (%)	SNR M1	SNR M2	Water SNR M1	Water SNR M2
8	331.42	155.91	32.11	27.04	1.64	1.03	10.32	5.76	931.99	476.54	201.60	150.79	19.53	26.15
6	301.98	137.74	37.52	28.21	1.64	1.03	8.05	4.88	704.87	388.14	183.69	133.21	22.82	27.29
4	248.83	116.04	38.83	29.23	1.64	1.03	6.41	3.97	540.86	296.97	151.35	112.23	23.62	28.27
2	166.46	81.56	43.83	31.39	1.64	1.03	3.80	2.60	279.82	159.84	101.25	78.88	26.66	30.36
1	122.56	57.86	50.96	34.72	1.64	1.03	2.40	1.67	140.49	66.63	74.55	55.96	31.00	33.58
0.5	57.77	36.14	56.67	38.42	1.64	1.03	1.02	0.94	1.93	-5.92	35.14	34.96	34.47	37.16

M1 and M2 correspond to two independent phantom measurements acquired on separate dates. Raw mean SI was obtained from the mean signal intensity of each phantom ROI in ImageJ. Water mean SI and Water SNR correspond to the position-matched water control acquired under the same sequence conditions for each extract concentration. Background noise SD was obtained from the standard deviation of a background ROI placed outside the phantom containers. A single background noise SD value was used for all phantom containers within the same sequence and independent measurement. Normalized SI was calculated as raw mean SI divided by the corresponding position-matched water mean SI. Signal enhancement (%) was calculated as [(normalized SI − 1) × 100]. SNR was calculated as raw mean SI divided by background noise SD. Water SNR was calculated as the corresponding position-matched water mean SI divided by the same background noise SD.

Table S2. Raw ROI signal intensity, position-matched water control values, background noise SD, normalized signal intensity, signal suppression, and SNR calculation for T2-weighted images of the *Ilex paraguariensis* aqueous extract.

Conc. (mg/mL)	Raw mean SI M1	Raw mean SI M2	Water mean SI M1	Water mean SI M2	Background noise SD M1	Background noise SD M2	Normalized SI M1	Normalized SI M2	Signal suppression M1 (%)	Signal suppression M2 (%)	SNR M1	SNR M2	Water SNR M1	Water SNR M2
8	45.81	25.56	290.24	258.97	2.82	0.61	0.16	0.09	84.22	90.13	16.27	41.97	103.07	425.24
6	74.5	46.94	333.00	273.50	2.82	0.61	0.22	0.17	77.76	82.84	26.30	77.07	118.25	449.10
4	135.57	85.78	367.00	281.87	2.82	0.61	0.37	0.30	63.06	69.57	48.14	140.86	130.33	462.85
2	277.09	146.12	430.29	288.19	2.82	0.61	0.64	0.51	35.60	49.30	98.40	239.94	152.80	473.22
1	274.09	181.68	470.00	305.09	2.82	0.61	0.58	0.59	41.68	40.45	97.34	298.34	166.90	501.00
0.5	389.95	222.00	484.19	325.16	2.82	0.61	0.80	0.68	19.46	31.73	138.48	364.53	171.94	533.92

M1 and M2 correspond to two independent phantom measurements acquired on separate dates. Raw mean SI was obtained from the mean signal intensity of each phantom ROI in ImageJ. Water mean SI and Water SNR correspond to the position-matched water control acquired under the same sequence conditions for each extract concentration. Background noise SD was obtained from the standard deviation of a background ROI placed outside the phantom containers. A single background noise SD value was used for all phantom containers within the same sequence and independent measurement. Normalized SI was calculated as raw mean SI divided by the corresponding position-matched water mean SI. Signal suppression (%) was calculated as [(1 − normalized SI) × 100]. SNR was calculated as raw mean SI divided by background noise SD. Water SNR was calculated as the corresponding position-matched water mean SI divided by the same background noise SD.

Table S3. Raw ROI signal intensity, position-matched water control values, background noise SD, normalized signal intensity, signal suppression, and SNR calculation for SSHTSE images of the *Ilex paraguariensis* aqueous extract.

Conc. (mg/mL)	Raw mean SI M1	Raw mean SI M2	Water mean SI M1	Water mean SI M2	Background noise SD M1	Background noise SD M2	Normalized SI M1	Normalized SI M2	Signal suppression M1 (%)	Signal suppression M2 (%)	SNR M1	SNR M2	Water SNR M1	Water SNR M2
8	5.95	4.29	486.36	418.82	4.24	1.61	0.01	0.01	98.78	98.97	1.40	2.67	114.73	260.46
6	7.59	5.13	603.36	459.96	4.24	1.61	0.01	0.01	98.74	98.88	1.79	3.19	142.34	286.05
4	15.09	14.39	741.72	487.47	4.24	1.61	0.02	0.03	97.97	97.05	3.56	8.95	174.98	303.15
2	167.87	98.25	909.97	548.39	4.24	1.61	0.18	0.18	81.55	82.08	39.60	61.10	214.67	341.04
1	228.93	201.20	1025.92	612.99	4.24	1.61	0.22	0.33	77.69	67.18	54.01	125.13	242.02	381.21
0.5	625.67	316.54	1156.48	699.50	4.24	1.61	0.54	0.45	45.90	54.75	147.60	196.86	272.82	435.01

M1 and M2 correspond to two independent phantom measurements acquired on separate dates. Raw mean SI was obtained from the mean signal intensity of each phantom ROI in ImageJ. Water mean SI and Water SNR correspond to the position-matched water control acquired under the same sequence conditions for each extract concentration. Background noise SD was obtained from the standard deviation of a background ROI placed outside the phantom containers. A single background noise SD value was used for all phantom containers within the same sequence and independent measurement. Normalized SI was calculated as raw mean SI divided by the corresponding position-matched water mean SI. Signal suppression (%) was calculated as [(1 − normalized SI) × 100]. SNR was calculated as raw mean SI divided by background noise SD. Water SNR was calculated as the corresponding position-matched water mean SI divided by the same background noise SD.

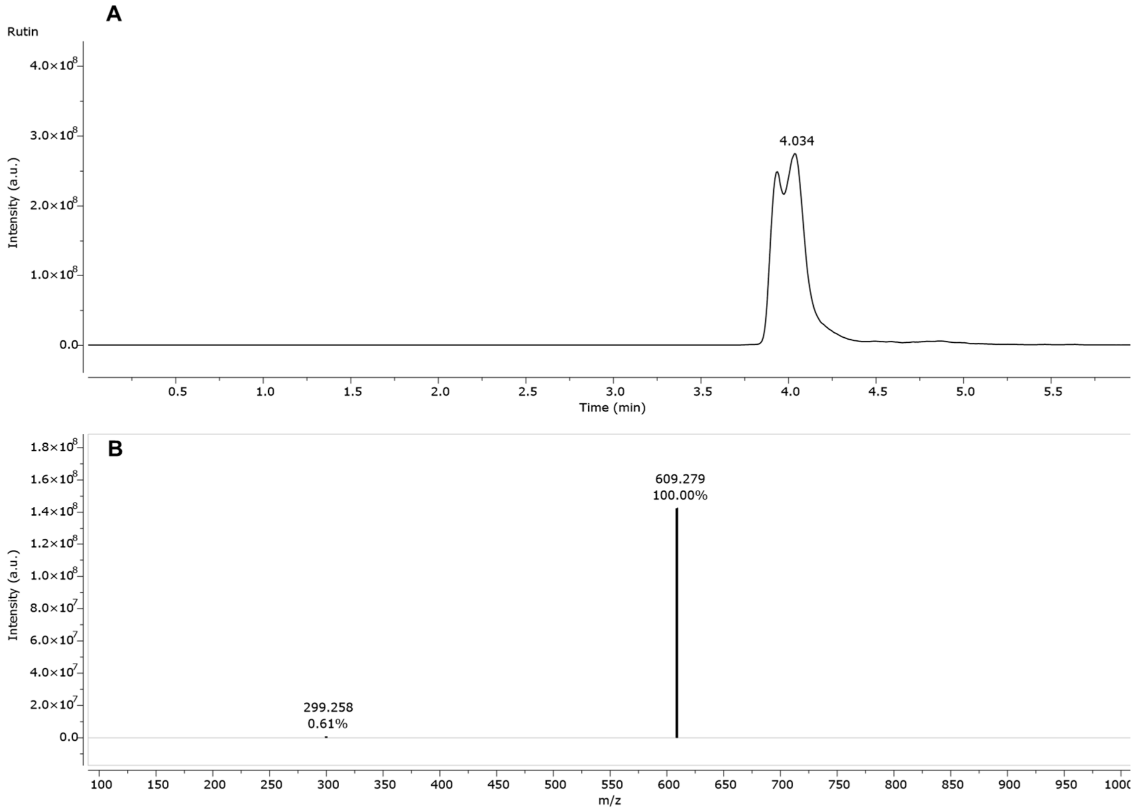

Figure S1. LC-ESI-MS/MS analysis of rutin analytical standard. (A) Chromatographic peak at Rt ≈ 4.03 min. (B) MS/MS spectrum showing a diagnostic fragment ion at m/z 609.30. This diagnostic fragment ion is show only for the rutin analytical standard and was not used as confirmatory evidence for compound identification in the *Ilex paraguariensis* aqueous extract.

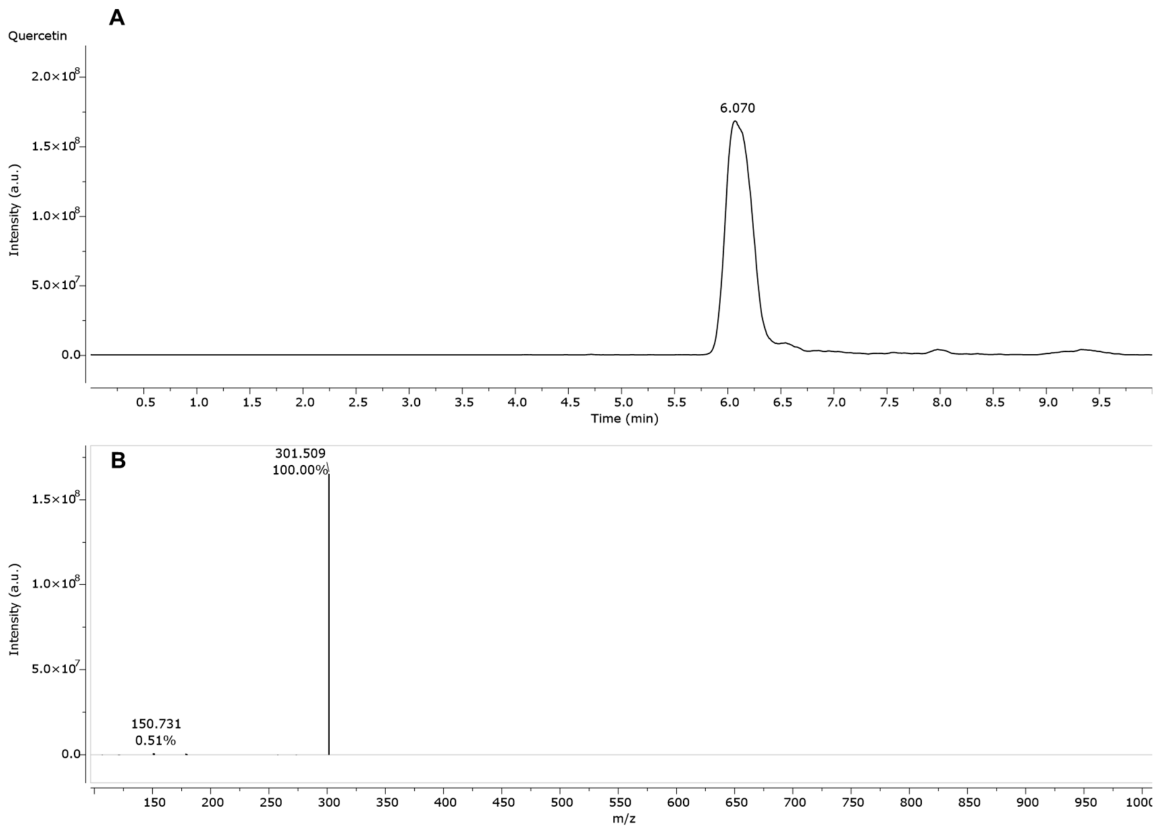

Figure S2. LC-ESI-MS/MS analysis of quercetin analytical standard. (A) Chromatographic peak at Rt ≈ 6.07 min. (B) MS/MS spectrum showing a diagnostic fragment ion at m/z 301.5. This diagnostic fragment ion is show only for the quercetin analytical standard and was not used as confirmatory evidence for compound identification in the *Ilex paraguariensis* aqueous extract.

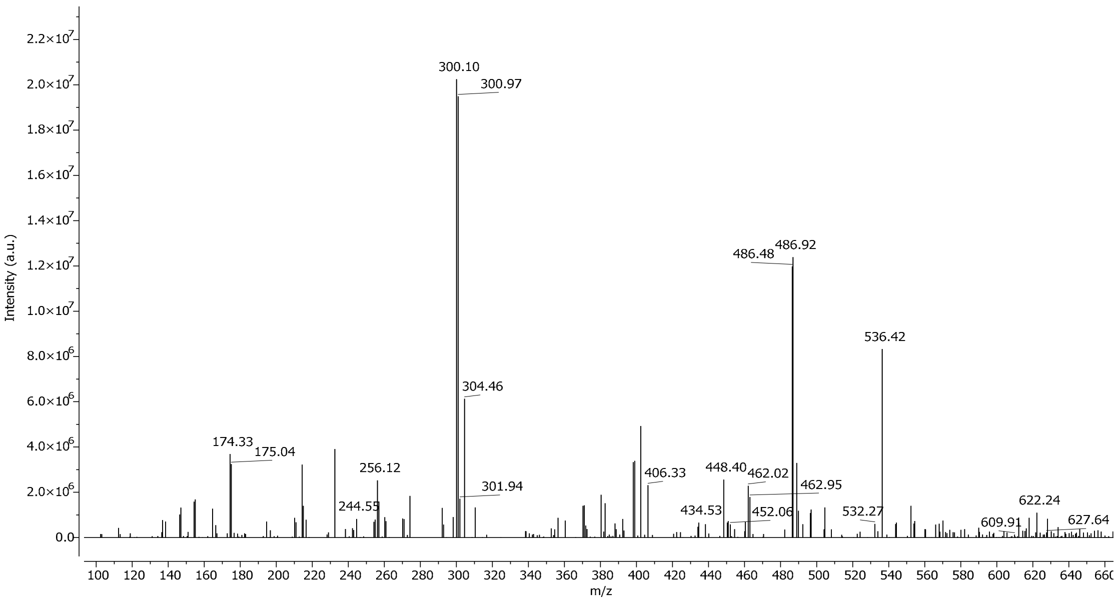

Figure S3. Mass spectrum (MS1) acquired at a retention time of 4.20 min within chromatographic Region A (apex at Rt ≈ 4.39 min) from the LC-ESI-MS analysis of the *Ilex paraguariensis* aqueous extract. The spectrum illustrates the nominal MS1 ionic profile associated with features eluting in this region. These signals should be interpreted as descriptive MS1 information and not as diagnostic fragment ions or definitive compound identification in the extract.

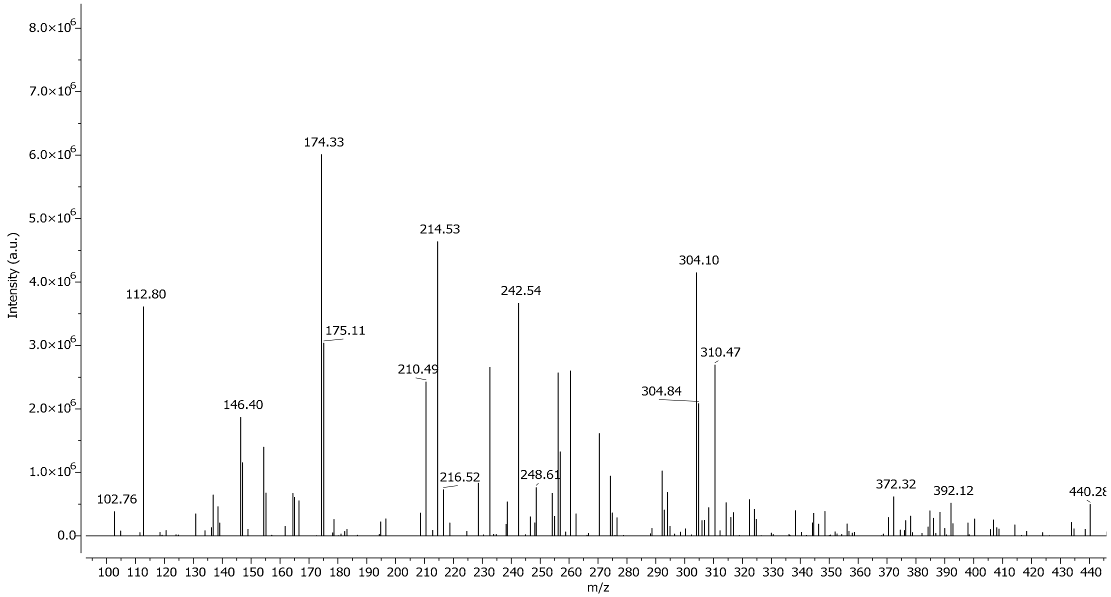

Figure S4. Mass spectrum (MS1) acquired at a retention time of 5.90 min (Region B) from the LC-ESI-MS analysis of the *Ilex paraguariensis* aqueous extract. The spectrum illustrates the nominal MS1 ionic profile and complexity of features eluting in this region. These signals should be interpreted as descriptive MS1 information and not as diagnostic fragment ions or definitive compound identification in the extract.
